# Construction of Xinjiang metabolic syndrome risk prediction model based on interpretable models

**DOI:** 10.1186/s12889-022-12617-y

**Published:** 2022-02-08

**Authors:** Yan Zhang, JAINA Razbek, Deyang Li, Lei Yang, Liangliang Bao, Wenjun Xia, Hongkai Mao, Mayisha Daken, Xiaoxu Zhang, Mingqin Cao

**Affiliations:** 1 Department of Epidemiology and Health Statistics, College of Public Health, Xinjiang Medical University, Urumqi, Xinjiang China; 2 Xinjiang De Kang Ci Hui Health Services Group, Urumqi, Xinjiang China

**Keywords:** Metabolic syndrome, Interpretable model, Prediction, Risk factors

## Abstract

**Background:**

We aimed to construct simple and practical metabolic syndrome (MetS) risk prediction models based on the data of inhabitants of Urumqi and to provide a methodological reference for the prevention and control of MetS.

**Methods:**

This is a cross-sectional study conducted in the Xinjiang Uygur Autonomous Region of China. We collected data from inhabitants of Urumqi from 2018 to 2019, including demographic characteristics, anthropometric indicators, living habits and family history. Resampling technology was used to preprocess the data imbalance problems, and then MetS risk prediction models were constructed based on logistic regression (LR) and decision tree (DT). In addition, nomograms and tree diagrams of DT were used to explain and visualize the model.

**Results:**

Of the 25,542 participants included in the study, 3,267 (12.8%) were diagnosed with MetS, and 22,275 (87.2%) were diagnosed with non-MetS. Both the LR and DT models based on the random undersampling dataset had good AUROC values (0.846 and 0.913, respectively). The accuracy, sensitivity, specificity, and AUROC values of the DT model were higher than those of the LR model. Based on a random undersampling dataset, the LR model showed that exercises such as walking (OR=0.769) and running (OR= 0.736) were protective factors against MetS. Age 60 ~ 74 years (OR=1.388), previous diabetes (OR=8.902), previous hypertension (OR=2.830), fatty liver (OR=3.306), smoking (OR=1.541), high systolic blood pressure (OR=1.044), and high diastolic blood pressure (OR=1.072) were risk factors for MetS; the DT model had 7 depth layers and 18 leaves, with BMI as the root node of the DT being the most important factor affecting MetS, and the other variables in descending order of importance: SBP, previous diabetes, previous hypertension, DBP, fatty liver, smoking, and exercise.

**Conclusions:**

Both DT and LR MetS risk prediction models have good prediction performance and their respective characteristics. Combining these two methods to construct an interpretable risk prediction model of MetS can provide methodological references for the prevention and control of MetS.

## Introduction

Metabolic syndrome (MetS) is a type of metabolic disorder characterized by central obesity, hypertension, hyperglycaemia and dyslipidaemia [1]. It is worth noting that the prevalence of MetS is on the rise due to rapid economic growth, an ageing population, lifestyle changes, and obesity. The prevalence of MetS in the world is approximately 20–25% [2], and the prevalence of MetS in China is approximately 13.6–46.3% [3–5]. Studies have shown that MetS is associated with an increased risk of diabetes, cardiovascular disease, cancer and even death [6]. MetS is an increasingly serious major public health problem and clinical challenge worldwide [7]. Therefore, appropriate prevention and control strategies must be adopted to reduce the incidence of MetS. Health checkups are the first stage of disease prevention, and data mining of medical checkup information can help identify people at high risk of MetS at an early stage, thus moving the timing of disease prevention and control forwards. The construction of MetS risk prediction models based on physical examination data is important for the prevention and control of MetS.

Disease risk prediction models predict the probability of disease occurrence by estimating the extent to which changes in one or more influencing factors affect health or disease [8] and are designed to identify people at high risk for a target disease so that effective interventions can be implemented before or early in the disease process. Therefore, the establishment of a MetS risk prediction model is of great practical importance for the early detection and intervention among people at high risk of MetS. The occurrence and development of MetS is the result of a combination of factors [9], including unchangeable factors such as sex, age and genetic history of the disease [10], and modifiable factors such as lifestyle, anthropometric indicators and blood parameters [11]. Among them, modifiable risk factors refer to factors that can be changed in principle. Accurately identifying modifiable risk factors for MetS and modifying them are effective measures to prevent and control MetS.

However, there are complex interactions between risk factors that do not facilitate the identification of MetS risk factors. Machine learning is an algorithm-based data analysis technology that has powerful data analysis capabilities and can effectively deal with complex relationships between variables. Considering these factors, this research constricted a risk prediction model for MetS based on machine learning methods.

Machine learning has proven to be effective in handling a large number of predictors while generating powerful predictive models to identify individuals at risk of developing diseases [12, 13]. Considering that medical field applications are more concerned about the transparency and interpretability of the models, we used two interpretable models of machine learning algorithms, logistic regression (LR) and decision tree (DT). LR uses equations to describe the relationship between input and target variables, which makes the prediction process simple. In DT, relying on a tree structure to classify samples and thus the interpretation of the prediction is pretty straightforward, using natural visualization [14, 15]. The classification process of DT starts from the root node, then searches from top to bottom along a branch, and finally uses the class label value of the leaf node as the class label to which the sample belongs.

In this paper, to establish a simple and practical risk prediction model for MetS, we built two interpretable models, LR and DT, based on easily available indicators such as demographic characteristics, anthropometric indicators, living habits, and family history of the subjects and then used a nomogram and tree diagram of the DT to explain and visualize the model. The interpretable MetS risk prediction model can help uncover risk factors, identify high-risk individuals, and provide methodological references for the prevention and control of MetS.

## Methods

### Research population

The data came from the health records database of the Dekang Cihui Medical Examination Center in Urumqi, Xinjiang Uygur Autonomous Region, China. A total of 37,457 medical examination reports were collected from January 2018 to December 2019. According to the exclusion criteria, we first deleted the data of 9199 subjects with missing diagnostic component data for MetS, then deleted 14 subjects younger than 18 years old, and finally had 25,542 as our research subjects. This study was approved by the Ethics Committee of the First Affiliated Hospital of Xinjiang Medical University, and all methods were carried out in accordance with relevant guidelines and regulations. The Ethics Committee of the First Affiliated Hospital of Xinjiang Medical University waived the need for informed consent.

### Data collection

All subjects needed to fast for 8 ~ 10 h before the health check-up. Data were collected by staff with uniform professional training. Demographic characteristics included the age and sex of the subjects. The anthropometric indices included height, weight, heart rate and blood pressure, and standard measuring instruments were used. Height and waist circumference were accurate to 0.1 cm, and weight was accurate to 0.1 kg. Body mass index (BMI) was calculated as weight in kilograms divided by height in metres squared. The blood pressure of the subjects in the sitting position was measured by using a mercury sphygmomanometer according to a standardized protocol. The subjects were asked to avoid strenuous exercise and drinking caffeinated beverages within 30 min before the measurement and to rest for at least 5 min before the first measurement, with an interval of 1 to 2 min between each measurement. Blood parameters included fasting blood glucose (FPG), triglycerides (TG), total cholesterol (TC), high-density lipoprotein cholesterol (HDL-C), and low-density lipoprotein cholesterol (LDL-C), which were measured by an automated chemistry analyser (Beckman Coulter chemistry analyser AU5800 series, Tokyo, Japan).

Questionnaire information included diabetes, hypertension, fatty liver, family history of hypertension, family history of diabetes, family history of coronary heart disease (CHD), family history of stroke, smoking (no-smoking meant never had smoking behaviour; smoking referred to smoking in the past 30 days at the time of investigation; quit smoking meant no longer smoking in the past 30 days at the time of the survey), drinking (no-drinking meant never drinking alcohol; sometimes drinking meant drinking less than 1 time per week in the past year; often drinking meant drinking ≥1 time/week in the past year; quit drinking meant no longer drinking in the past 30 days), exercise, and eating habits.

### Diagnostic Criteria for MetS

Using the diagnostic criteria recommended by the Chinese Diabetes Society [16], if at least three of the following components are present, the patient is diagnosed as MetS: ① Overweight and/or obesity: BMI≥25; ② Hyperglycaemia: fasting blood glucose ≥ 6.1 mmol/L, and/or those who have been diagnosed with diabetes and treated; ③ Hypertension: systolic/diastolic blood pressure ≥140/90 mmHg, and/or those who have been diagnosed with hypertension and treated; ④ Dyslipidaemia: TG≥1.7 mmol/L, and/or HDL-C <0.9 mmol/L (male), <1.0 mmol/L (female).

### Data preprocessing

There were some missing data in the dataset, and the amount of missing data could not be ignored, so data filling was needed. Multiple imputation (MI) is a popular method to address missing data and has the characteristics of flexibility and robustness [17]. Compared with other methods, MI has certain advantages in dealing with missing data [18]. Therefore, MI was used to fill the missing data in the study to make full use of the dataset. We randomly split the dataset (n = 25,542) into a training dataset (70%, n = 17,879) and a test dataset (30%, n = 7663) according to a 7:3 ratio. Among the subjects in the training dataset, there were 15,599 (87.2%) nonmetabolic syndrome (non-MetS) and 2280 (12.8%) MetS patients. In the test dataset, there were 6,676 (87.1%) non-MetS and 987 (12.9%) MetS patients. The imbalance ratio of the training dataset and test dataset was 7:1. Studies have shown that in an imbalanced dataset, if a small number of classes are used as the research target, the sensitivity of building a model is usually very low [19], which is a difficult problem for researchers.

Resampling technology is a useful preprocessing step to solve imbalanced problems [20]. It can modify the imbalance distribution of the majority and minority classes at the data level before training with classifiers. For most imbalanced datasets, the application of resampling technology improved the performance of the classifier [21]. Resampling technology is mainly divided into oversampling, undersampling and hybrid sampling [22]. Two commonly used and effective methods for oversampling are random oversampling and the synthetic minority oversampling technique (SMOTE). Random oversampling adds new samples by randomly copying samples in the minority class. SMOTE analyses minority samples and artificially synthesizes new samples based on the minority samples to add to the dataset [23]. Random undersampling randomly removes samples from the majority class until both classes are equally balanced. Hybrid sampling oversamples minority samples with replacement and undersamples the majority samples without replacement to balance the class distribution. Considering that these four methods have their own characteristics, this study used random oversampling, SMOTE, random undersampling, and hybrid sampling to process the unbalanced training dataset.

### Risk prediction model

#### Logistic regression

LR is a probabilistic nonlinear regression model that can analyse the relationship between one or more factors (independent variables) and observations (dependent variables). The dependent variable can be a categorical variable or a rank variable. LR has advantages in interpreting model results and calculation costs [24]. Let P(y = 1|X) represent the probability of an individual’s onset when the exposure factor is X, and the ratio of the probability of onset *P* to the probability of not onset 1-*P* is the odds; then, logit*P* is the log of odds:

$${log}itP=ln\left(\frac{P}{1-P}\right)$$

LR model is:

$${log}itP={\beta }_{0}+{\beta }_{1}{x}_{1}+{\beta }_{2}{x}_{2}+\cdots +{\beta }_{m}{x}_{m}$$

The constant term ${\beta }_{0}$ represents the natural logarithm of the ratio of the individual’s onset and nononset probability when the exposure dose is 0, and the regression coefficient ${\beta }_{j}(j=\text{1,2},\cdots ,m)$ represents the amount of change in logit*P* when the independent variable changes by one unit.

The nomogram is based on the LR model, which integrates multiple predictors and uses scaled lines drawn on the same plane at a certain scale to express the interrelationships among the variables in the prediction model. It transforms complex regression equations into visual graphs so that the contribution of the predictor variables to the outcome can be reflected visually and directly [25]. Therefore, this study established a nomogram to evaluate the risk of MetS in an intuitive and easy-to-understand manner.

#### Decision tree

DT is a nonparametric method that has many advantages, including dealing with the nonlinear relationship between variables and low computational overhead, and the model results are easy to interpret [26], so it is widely used in various fields. However, DT learning algorithms may produce models that are overly complex and/or biased if the data are imbalanced [27]. The C4.5 decision tree is the most commonly used DT algorithm, which inherits all of the advantages of the ID3 algorithm and improves on it. The C4.5 algorithm uses a top-down recursive method and uses the information gain rate as the criterion for selecting branch attributes. Assuming that the sample set is *S*, the sample attribute $A$ has $\nu$ possible values; that is, the sample set S can be divided into $\nu$ subsample sets$\left\{{S}_{1},{S}_{2}\cdots {S}_{\upsilon }\right\}$ through attribute A, and $Gain\left(S,A\right)$ is the information gain corresponding to attribute A. The information gain rate $Gain\_Ratio$ of attribute A is defined as:

$${SplitInfo}_{A}\left(S\right)=-\sum _{j = 1}^{\nu }\frac{\left|{S}_{j}\right|}{\left|S\right|}\times {log}_{2}\left(\frac{\left|{S}_{j}\right|}{\left|S\right|}\right)$$

$$Gain\_Ratio\left(A\right)=Gain\left(A\right)/SplitInfo\left(A\right)$$

### Statistical analysis

Data were preprocessed and analysed using R statistical software (version 3.6.0, http://www.r-project.org). Baseline characteristics of the subjects included in the study were described statistically, quantitative variables were described using the mean and standard deviation, and qualitative variables were described using cases and percentages. The baseline characteristics of subjects with MetS and non-MetS were compared based on the type and characteristics of the data, the quantitative variables were compared using independent sample t-tests, the categorical variables were compared using chi-square tests, and the ranking data were compared using the Kruskal–Wallis H test. The training dataset was used to train the model, and the test dataset was used to evaluate the effect of the model. The training dataset was resampled using the ROSE package, and then the LR and DT models were trained using the rms package and the party package, respectively. The Hosmer-Lemeshow goodness‐of‐fit test was used to evaluate the model calibration by comparing the observed and predicted probabilities. A value of *P*_HL_ >0.05 indicated satisfactory calibration. The prediction performance of the model was evaluated by the area under the receiver operating characteristic curve (AUROC), sensitivity, specificity, and accuracy. Among them, the AUROC value is not affected by the data imbalance and reflects the objective [28]. Their values are between 0 ~ 1, and the closer the value is to 1, the better the model classification accuracy. The AUROC values and 95% CIs of the models were calculated and compared using MedCalc statistical software (version 15.6.1, https://www.medcalc.org). Two-tailed *P* < 0.05 was considered statistically significant.

## Results

### Baseline characteristics

Of the 25,542 participants included in the study, 3,267 (12.8%) were diagnosed with MetS, and 22,275 (87.2%) were diagnosed with non-MetS. A comparison of baseline characteristics between MetS and non-MetS patients is shown in Table 1. The MetS and non-MetS groups had statistically significant baseline characteristics, including sex, age, heart rate, previous diabetes, previous hypertension, fatty liver, smoking, drinking, hypertension family history, family history of diabetes, exercise, eating habits, BMI, SBP, and DBP (*P *< 0.05).

**Table 1:** Comparison of baseline characteristics between MetS and Non-MetS (*n* (*n*%) / $\overline{x}$ ± s)

**Variable**	**Non-MetS (n = 22,275)**	**MetS (n = 3267)**	$t/{\chi }^{2}/H$	***P*** **value**
Gender				
Male	10,897(82.7)	2282(17.3)	499.737	< 0.001
Female	11,378(92.0)	985(8.0)		
Age (years)				
18 ~ 44	13,696(93.0)	1023(7.0)	1220.867	< 0.001
45 ~ 59	6193(81.9)	1371(18.1)		
60 ~ 74	1963(72.6)	741(27.4)		
75 ~ 89	423(76.2)	132(23.8)		
Heartrate	68.88 ± 9.15	73.30 ± 10.87	-22.088	< 0.001
Previous diabetes				
No	21,858(89.8)	2487 (10.2)	3088.048	< 0.001
Yes	417(34.8)	780(65.2)		
Previous hypertension				
No	20,792(91.0)	2060(9.0)	2773.785	< 0.001
Yes	1483(55.1)	1207(44.9)		
Fatty liver				
No	17,341(93.8)	1137(6.2)	2638.776	< 0.001
Yes	4934(69.8)	2130(30.2)		
Smoking				
No	15,035(90.1)	1653(9.9)	415.572	< 0.001
Quit	997(75.4)	325(24.6)		
Yes	6243(82.9)	1289(17.1)		
Drinking				
No	8642(87.9)	1185(12.1)	234.245	< 0.001
Quit	164(78.5)	45(21.5)		
Sometimes	12,362(88.1)	1664(11.9)		
Often	1107(74.8)	373(25.2)		
Family history of hypertension				
No	13,504(88.6)	1745(11.4)	61.580	< 0.001
Yes	8771(85.2)	1522(14.8)		
Family history of diabetes				
No	18,766(87.5)	2676(12.5)	11.547	0.001
Yes	3509(85.6)	591(14.4)		
Family history of CHD				
No	20,356(87.2)	2994(12.8)	0.243	0.622
Yes	1919(87.5)	273(12.5)		
Family history of stroke				
No	22,019(87.2)	3230(12.8)	0.007	0.933
Yes	256(87.4)	37(12.6)		
Exercise				
Hardly	9272(88.3)	1227(11.7)	116.049	< 0.001
Walk	9049(84.7)	1631(15.3)		
Run and others	3954(90.6)	409(9.4)		
Eating habits				
Light	3065(88.3)	406(11.7)	29.005	< 0.001
General	12,242(87.8)	1703(12.2)		
Sweet	274(87.3)	40(12.7)		
Salty	6390(85.9)	1051(14.1)		
Meat	304(81.9)	67(18.1)		
BMI (kg/m^2^)				
<18.5	1060(100.0)	0(0.0)	4267.304	< 0.001
18.5 ~ 23.9	11,168(99.0)	110(1.0)		
24 ~ 26.9	6444(84.4)	1192(15.6)		
27 ~ 29.9	2690(67.8)	1277(32.2)		
≥30	913(57.0)	688(43.0)		
SBP (mmHg)	121.49 ± 17.80	146.62 ± 16.44	-80.725	< 0.001
DBP (mmHg)	75.63 ± 10.77	90.57 ± 10.74	-74.235	< 0.001

*SBP* systolic blood pressure; *DBP* diastolic blood pressure

### Building risk prediction models

#### Comparing model classification performance

**Fig. 1:**
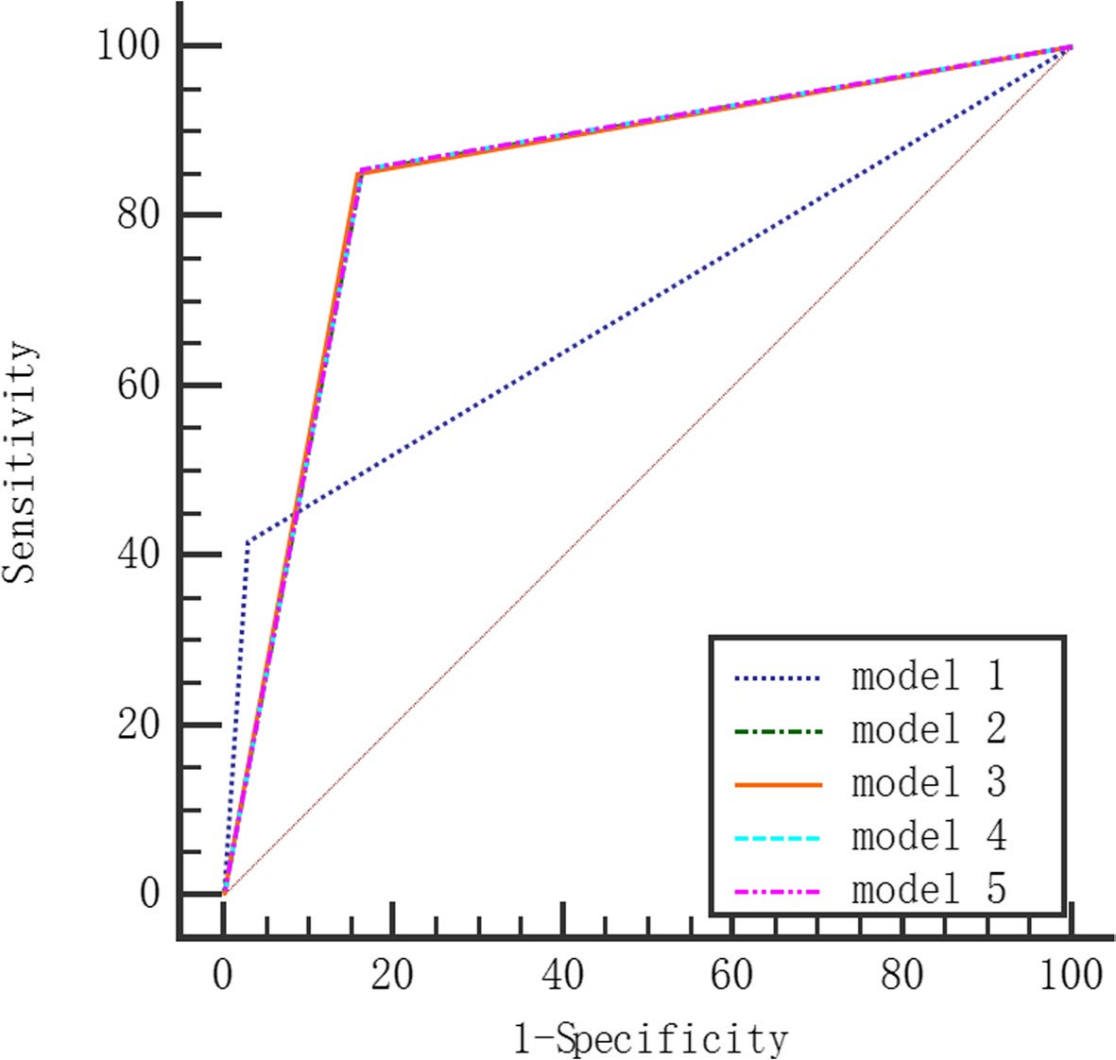
ROC curve of LR model

**Fig. 2:**
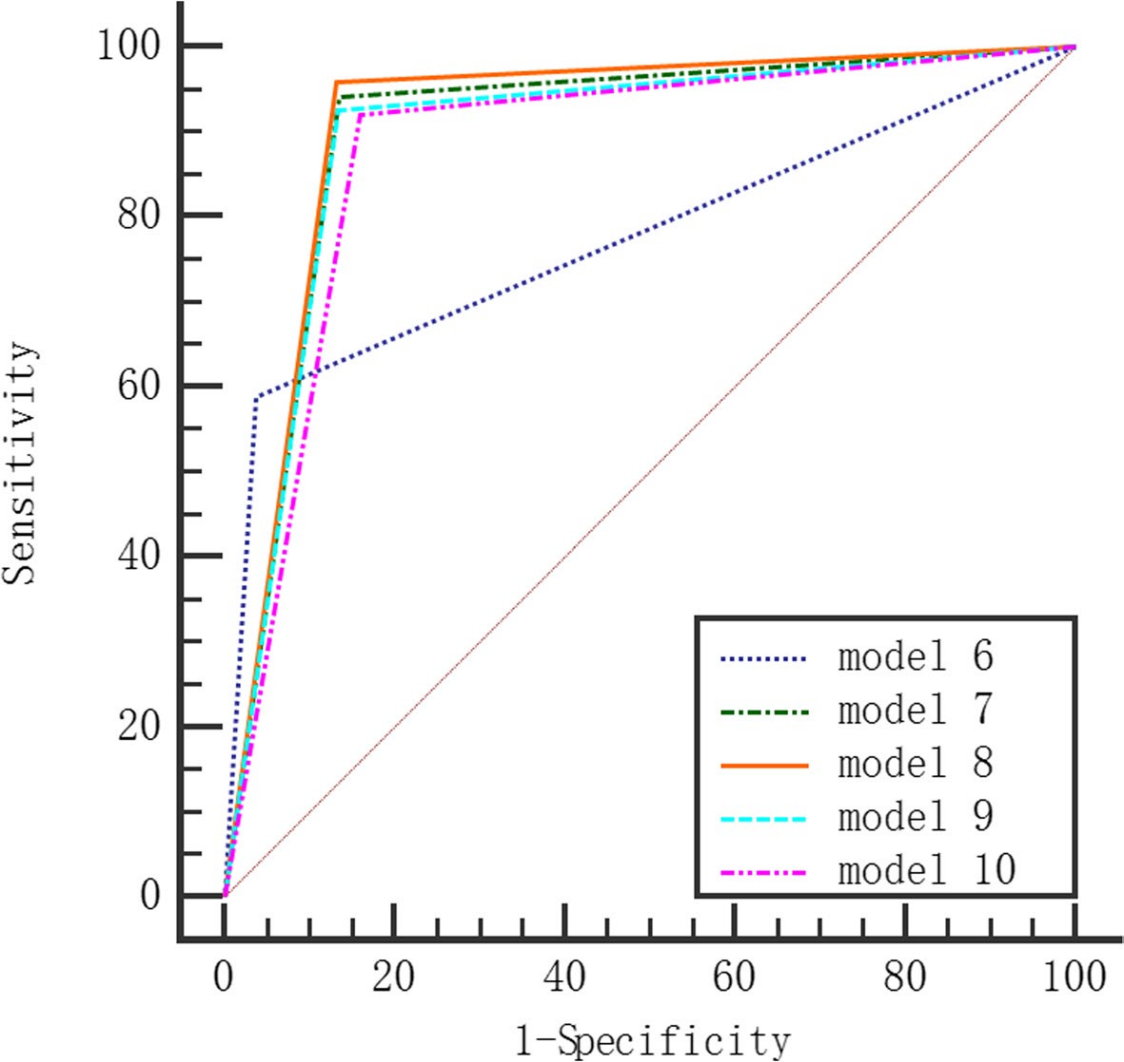
ROC curve of DT model

We selected statistically significant variables for LR (Model 1 ~ Model 5) and DT (Model 6 ~ Model 10) multivariate analysis based on five datasets: original imbalanced training dataset, random oversampling, random undersampling, hybrid sampling, and SMOTE. The comparison of the classification performance of each model on the test dataset is shown in Table 2. Compared with the original dataset, the random oversampling, random undersampling, hybrid sampling and SMOTE datasets had decreased accuracy and specificity on LR and DT but increased sensitivity and AUROC values. Both LR and DT models based on random undersampling datasets had better AUROC values. The accuracy, sensitivity, specificity, and AUROC values of the DT model were higher than those of the LR model. The ROC curves of the LR and DT models are shown in Figs. 1 and 2.

**Table 2:** Classification performance comparison between the DT and LR models

Model	Datasets	Accuracy	Sensitivity	Specificity	AUROC (95% Cl)
LR					
Model 1	Original imbalanced	0.901	0.418	0.971	0.694(0.684 ~ 0.704)
Model 2	Random oversampling	0.839	0.854	0.837	0.846^*^(0.837 ~ 0.854)
Model 3	Random undersampling	0.843	0.851	0.842	0.846^*^(0.838 ~ 0.854)
Model 4	Hybrid sampling	0.839	0.856	0.837	0.847^*^(0.838 ~ 0.855)
Model 5	SMOTE	0.838	0.855	0.836	0.846^*^(0.837 ~ 0.854)
DT					
Model 6	Original imbalance	0.915	0.588	0.962	0.775(0.766 ~ 0.785)
Model 7	Random oversampling	0.874	0.942	0.864	0.903^#^(0.896 ~ 0.910)
Model 8	Random undersampling	0.879	0.959	0.868	0.913^#^(0.907 ~ 0.919)
Model 9	Hybrid sampling	0.873	0.926	0.866	0.896^#^(0.889 ~ 0.902)
Model 10	SMOTE	0.851	0.920	0.841	0.880^#^(0.873 ~ 0.888)

* *P* <0.05 compared with the AUROC value of Model 1. # *P* <0.05 compared with the AUROC value of Model 6.

#### Logistic regression model

The LR model based on a random undersampling dataset was used to analyse the factors influencing MetS, as shown in Table 3. The LR model Hosmer-Lemeshow good of fit test χ^2^ = 10.691, *P*=0.138, showed that the model fits well. The results showed that age, previous diabetes, previous hypertension, fatty liver, smoking, exercise, SBP and DBP were all associated with MetS (*P *< 0.05). Among these factors, exercises such as walking (OR=0.769) and running (OR= 0.736) were protective factors for MetS, but age 60 ~ 74 years (OR=1.388), previous diabetes (OR=8.902), previous hypertension (OR=2.830), fatty liver (OR=3.306), smoking (OR=1.541), high systolic blood pressure (OR=1.044), and high diastolic blood pressure (OR=1.072) were risk factors for MetS.

**Table 3:** Logistic regression analysis of influencing factors of MetS

Variable	Coefficients	Std Error	Wald	OR	95% *CI*	*P* value
Intercept	-12.744	0.437	-29.152	-	-	-
Age (reference: 18 ~ 44)						
45 ~ 59	0.139	0.098	1.418	1.149	0.948 ~ 1.391	0.156
60 ~ 74	0.328	0.141	2.320	1.388	1.052 ~ 1.833	0.020
75 ~ 89	-0.117	0.274	-0.427	0.890	0.522 ~ 1.531	0.669
Previous diabetes (reference: No)						
Yes	2.186	0.178	12.281	8.902	6.333 ~ 12.739	<0.001
Previous hypertension (reference: No)						
Yes	1.040	0.121	8.619	2.830	2.238 ~ 3.593	<0.001
Fatty liver (reference: No)						
Yes	1.196	0.085	14.053	3.306	2.800 ~ 3.908	<0.001
Smoking (reference: No)						
Quit	0.325	0.169	1.925	1.384	0.996 ~ 1.930	0.054
Yes	0.432	0.091	4.731	1.541	1.288 ~ 1.844	<0.001
Exercise (reference: Hardly)						
Run and others	-0.306	0.125	-2.451	0.736	0.576 ~ 0.940	0.014
Walk	-0.262	0.096	-2.734	0.769	0.637 ~ 0.928	0.006
SBP	0.044	0.004	11.605	1.044	1.037 ~ 1.052	<0.001
DBP	0.070	0.006	11.497	1.072	1.060 ~ 1.085	<0.001

**Fig. 3:**
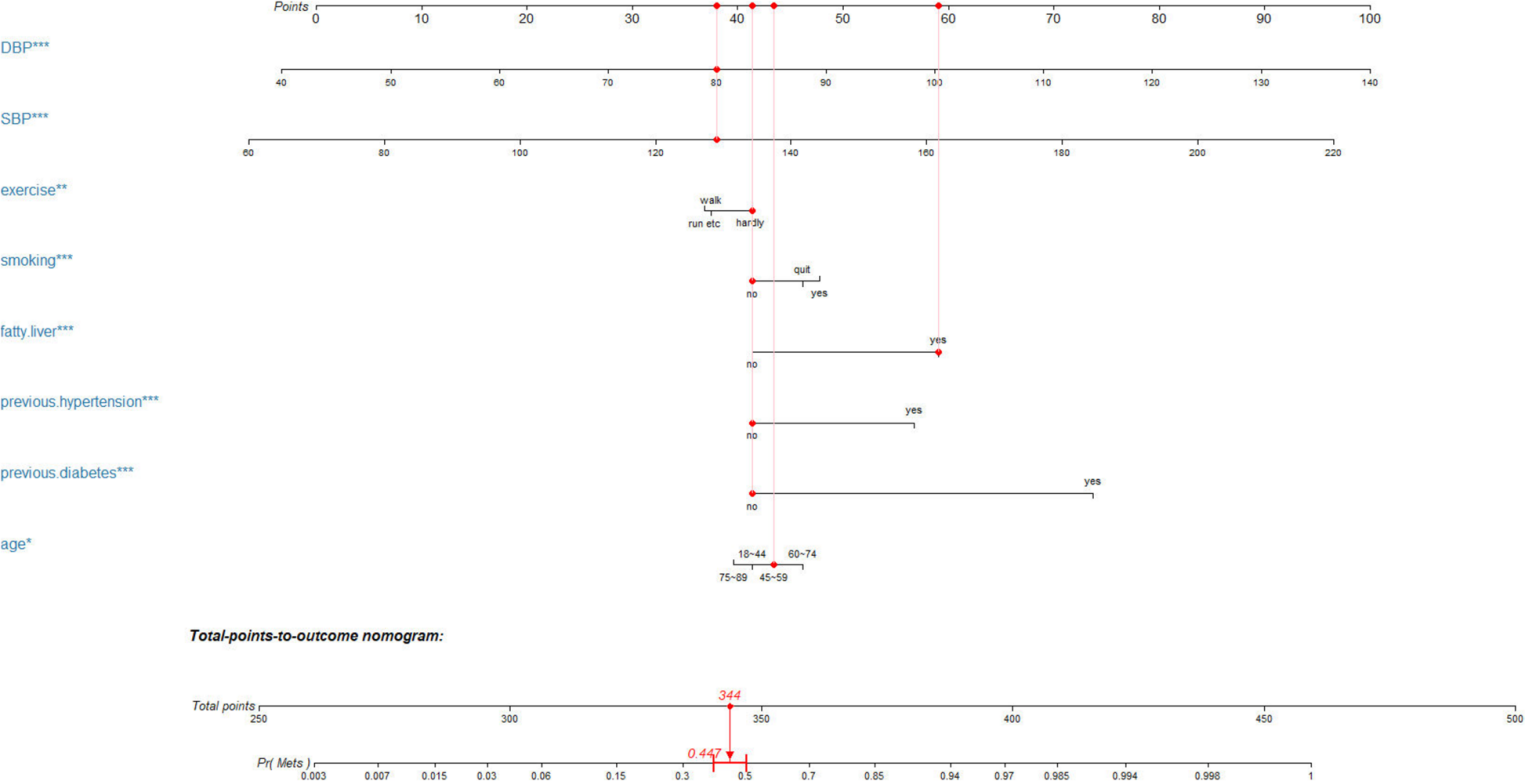
Nomogram of risk prediction for MetS

Figure 3 shows the nomogram of MetS risk prediction based on the LR model. First, the score points corresponding to each variable value were found, then all of the score points were summed to obtain the total score, and finally, the corresponding probability of MetS was determined. As an example, a person was 50 years old, the corresponding score was 43; no previous diabetes, the corresponding score was 41; no previous hypertension, the corresponding score was 41; fatty liver, the corresponding score was 59; no smoking, the corresponding score was 41; there was almost no exercise, the corresponding score was 41; the SBP was 129 mmHg, the corresponding score was 40; the DBP was 80 mmHg, the corresponding score was 38; the total score was 344, so the probability of this person having MetS was 0.451.

#### Decision tree model

**Fig. 4:**
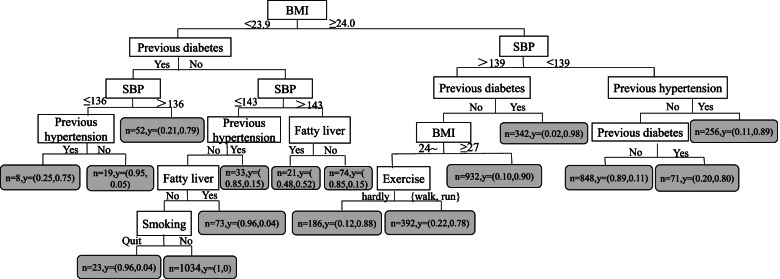
Decision tree model of influencing factors of MetS

The DT model, which had 7 depth layers and 18 leaves and was based on the random undersampling dataset, is shown in Fig. 4. BMI, as the root node of the DT, was the most important factor affecting MetS. The other variables are ranked in descending order of importance: SBP, diabetes, hypertension, DBP, fatty liver, smoking, and exercise.

## Discussion

This study found that the prediction performance of the two classification models, LR and DT, was poor due to imbalanced datasets. However, the predictive performance of the classification model was significantly improved after balancing the dataset using random oversampling, SMOTE, random undersampling, and hybrid sampling, which indicated that resampling technology played an important role in improving the performance of the models on the imbalanced datasets. Among the four resampling methods, random undersampling had the best performance, which was different from the viewpoints of some previous studies [29–31]. Related studies found that random oversampling and SMOTE had better performances. We believe a possible reason is that the characteristics of the study data satisfied the conditions for the applicability of random undersampling. Random undersampling is suitable for a case where the number of samples of the majority class is very large, and the dataset is balanced by reducing the size of the redundant classes. In our study, 22,275 MetS subjects belonged to the majority class, and the larger sample size satisfied the application conditions of the random undersampling method. Therefore, random undersampling was the best method for balancing datasets in our research.

The two MetS risk prediction models constructed in this study, LR and DT, both had better prediction performance with AUROC values of 0.846 and 0.913, respectively. However, for applications, not only the prediction performance of the risk prediction model but also the interpretability of the model is of concern. Interpretation of risk prediction models, even the simplest ones, is not straightforward for clinicians and their patients [32]. Previous studies have shown that patients prefer graphical representations of risks over digital risk estimates because this can improve their understanding of risks [33]. In this study, two visualization methods of the nomogram and tree diagrams of DT were used to show the risk of MetS occurrence, which are convenient for understanding the model results. We found that the results of the LR and DT models were basically consistent, both indicating that previous diabetes, previous hypertension, fatty liver, smoking, exercise, SBP and DBP are risk predictors for MetS. Notably, BMI, hypertension, diabetes, blood pressure, glucose, and lipid levels are already conventionally used in the diagnosis of MetS, and all have been shown to be risk factors for MetS. However, there were some differences in the results of the two models. The DT model showed that BMI was a very important risk predictor for MetS. Similarly, Jeong H S et al. also found that BMI was an important predictor of MetS [34]. In addition, the LR model showed that age was one of the risk predictors of MetS, and the risk of MetS in subjects aged 60 ~ 74 years old was 1.388 times that of 18 ~ 44 years old, which was similar to the findings of Wang S et al. [35]. Since both age and BMI are widely considered to be risk factors for MetS, if we only focus on the results of one model, it will lead to ignoring another important factor, so by combining LR and DT models, we can better identify risk factors for MetS.

The study results showed that DT had better predictive performance than LR on the original unbalanced and the balanced datasets. Thus, DT had a better prediction performance and ability to handle unbalanced data, which is similar to the research results of Sankari E S et al. [36]. DT is one of the interpretable models in machine learning that can lead to a simple, clear, and intuitive tree structure to show the meaningful classification of prediction variables [37], eliminate the impact of collinearity between variables, and visually show the interactions between variables. In addition, it is of interest to us that the tree model can be used to generate easy-to-understand rules to guide the prevention and control of MetS. Currently, DT models have been applied in clinical practice, including disease diagnosis, risk of disease occurrence, regression and prognosis [38–40], health economics evaluation [41], clinical decision support system (CDSS) [42], and rational and safe drug use [43], and DT has shown powerful performance and high accuracy in all these aspects. Although studies have shown that the prediction performance of LR is lower than that of the DT, LR is not prone to overfitting and has good generalization ability [44].

Therefore, the MetS risk prediction model based on LR has a certain population promotion ability. In addition, since the LR model includes eight easily available indicators of age, SBP, DBP, exercise, smoking, fatty liver, hypertension and diabetes, the nomogram we developed on this basis has the advantages of being simple, intuitive and practical, can be used as a scoring tool for predicting the risk of MetS, and has some auxiliary value in clinical applications.

This study used the data preprocessing method resampling technique to deal with the data class imbalance problem. This study not only constructed the MetS risk prediction model based on two interpretable models, LR and DT, both with good prediction performance, but also used two visualization methods, nomograms and tree diagrams of DT, to present the model results. However, the MetS risk prediction models were developed and internally validated based on a physical examination population and we did not conduct external validation of the models. In addition, because this was a cross-sectional study, any associations observed in this study do not imply causal relationships.

## Conclusions

The two interpretable models, DT and LR, have their own characteristics, and their combined use is complementary. Combining the two methods to construct an interpretable risk prediction model of MetS can provide methodological references for the prevention and control of MetS.

## Data Availability

The datasets used and analysed during the current study are not publicly available due to the need to protect the individual privacy of the patients included in this study, but they are available from the corresponding author upon reasonable request.

## Abbreviations

MetS: metabolic syndrome
non-MetS: nonmetabolic syndrome
BMI: body mass index
DBP: diastolic blood pressure
SBP: systolic blood pressure
OR: odds ratio
CI: confidential interval
AUC: area under the curve
CHD: coronary heart disease
DT: decision tree
LR: logistic regression
AUROC: area under the receiver operating characteristic curve
ROC curve: receiver operating characteristic curve
